# Surface Wave Cloak from Graded Refractive Index Nanocomposites

**DOI:** 10.1038/srep29363

**Published:** 2016-07-15

**Authors:** L. La Spada, T. M. McManus, A. Dyke, S. Haq, L. Zhang, Q. Cheng, Y. Hao

**Affiliations:** 1 School of Electronic Engineering and Computer Science, Queen Mary University of London, E1 4NS, England, UK; 2 BAE Systems Advanced Technology Centre, BS34 7QW, England, UK

## Abstract

Recently, a great deal of interest has been re-emerged on the possibility to manipulate surface waves, in particular, towards the THz and optical regime. Both concepts of Transformation Optics (TO) and metamaterials have been regarded as one of key enablers for such applications in applied electromagnetics. In this paper, we experimentally demonstrate for the first time a dielectric surface wave cloak from engineered gradient index materials to illustrate the possibility of using nanocomposites to control surface wave propagation through advanced additive manufacturing. The device is designed analytically and validated through numerical simulations and measurements, showing good agreement and performance as an effective surface wave cloak. The underlying design approach has much wider applications, which span from microwave to optics for the control of surface plasmon polaritons (SPPs) and radiation of nanoantennas.

Transformation Optics (TO)^1^ is a topic of great interest in the last few decades. TO allows a link between geometries and electromagnetic wave behavior to be formed. At the heart of this concept is the invariance in form of Maxwell’s equations when transforming from one space to another. One of exemplar designs from the concept of TO is to design invisibility cloaks, which is used to effectively render the object from impinging electromagnetic waves. A number of novel approaches, for free space waves, have been proposed over the years to accomplish this based on, for example, the scattering cancellation approach^2^ by using homogeneous isotropic layer of material with permittivity below unity (plasmonic cloaking)^3^ or ultrathin isotropic frequency selective surfaces (mantle cloak)^4^; the transmission-line network method^5^; the parallel-plate^6^ and active materials^7^. Unfortunately, all such approaches suffer of several drawbacks: they are dependent on the geometry and shape of the object to cloak, not very suited for electrically large dimensions, rely on material properties not easily found in nature and they are extremely sensible to material losses and polarization dependent.

Although during these past years several studies focused their attention to free space wave, primarily from three-dimensional^8^,^9^, non-Euclidean transformations^10^, the quasi-optical^11^ and the combined carpet cloak-metasurface approach^12^. Relatively little studies are present in the area of surface waves cloaking. However there are significant technological and industrial interests in exploring waves traveling on materials surface. Recently, the need to understand the mechanisms of surface waves, and the ability to control and tailor their propagation characteristics, arise. The importance of waves confined or associated with surfaces, cannot be underestimated as they are keys to developing solutions for reducing and mitigating important issues such as: the scattering from surfaces, re-radiation from practical features such as steps, gaps and engineering elements that exist on real-life platforms. Without this understanding it is not possible to take a robust approach to design materials and, in turn, have significant impact on the ability to design platforms, with specific required electromagnetic properties.

The aim of this paper is therefore to demonstrate the TO design for surface waves through the first implementation of a fully dielectric surface wave cloak from the combination of curved geometries and graded index media. The device is analytically, numerically and experimentally validated in the frequency range 8–10 GHz, showing very good and promising performances for an effective surface wave cloak.

## Methods

### Analytical design for surface wave

**Figure 1:**
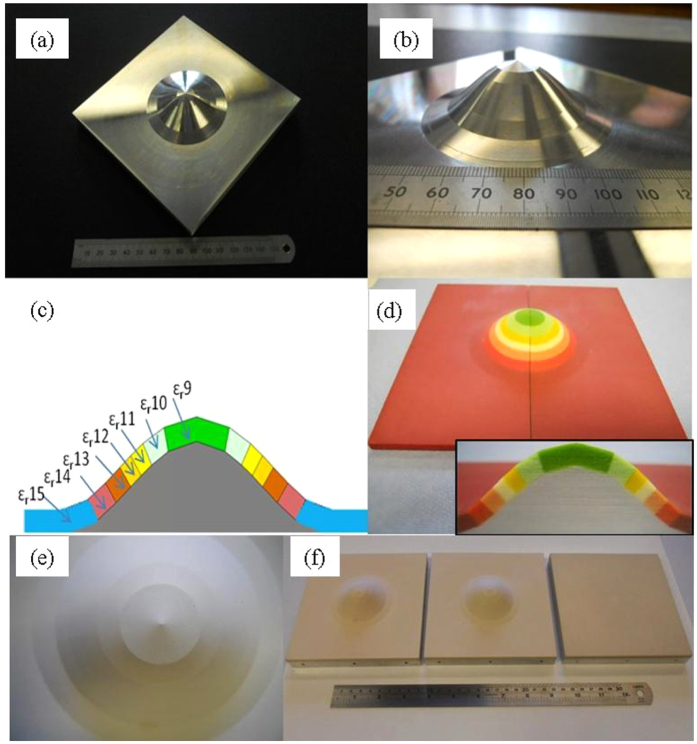
Cosine-shaped surface deformation: (**a**) top-view and (**b**) side-view; Schematic indicating the required permittivity values for each layer (**c**); 3D printed prototype of the cloak structure with cross-section inset (**d**); Fabricated surface wave structures: (**e**) plane view of the samples and (**f**) the three composite structures manufactured.

We recently presented in ref. 13 a theoretical framework to manipulate surface wave via TO for a rotationally symmetric curved surface on which waves can propagate (Fig. 1(a,b)). For the case of “surface wave cloaks”, a dielectric profile with graded refractive indices can be achieved to ensure that the wave propagation on a curved surface can emulate that over a flat surface one. We refer to a spherical coordinate system (*r*, *θ*, *ϕ*). The permittivity value is calculated by equating the optical path length of the ray, crossing the flat plane with homogeneous permittivity, to the optical path on the rotationally symmetric curved surface with permittivity that depends on the angle, for two orthogonal paths: the circular one (of fixed radius), and the second one: the radial path (of fixed angle). After some mathematical manipulations the final expression reads^13^:


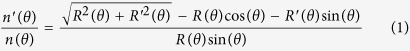


The formula proposed is a general tool to design any surface wave device with rotationally symmetric profiles. For the instance of surface wave cloaks, the obtained permittivity profile ensures that the surface wave propagating on a curved space, behaves as it travels along a flat homogeneous plane.

To describe the surface wave behaviour analytically, we start with the Helmholtz Differential Equation (HDE) for the electric field ∇^2^E + *k*^2^E = 0.

By using separation of variables, the total electric field can be evaluated in a closed form formula as follow^14^:


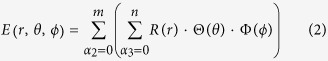


where *R*(*r*), Θ(*θ*), Φ(*ϕ*) are the radial, the angular and azimuthal components, respectively, *m* and *n* integers.

### The isotropic all-dielectric structure implementation

By using the aforementioned design process, a practical application of the structure is here presented. In particular, a surface wave implementation using a graded dielectric slab above a ground plane is studied. To achieve the appropriate propagation characteristics, it is necessary to alter the permittivity of the materials accordingly, for a given slab thickness, so that the effective refractive index achieves the required value^13^.

The related refractive index distribution is shown in Fig. 1(c,d), for a 4.5 mm slab. The permittivity profile chosen to cloak the rotational symmetric cosine surface was chosen in the range 9–15 for two reasons:

- Reliable control of the surface wave: confinement is crucial for cloaking applications, to ensure minimal radiation from the surface when curved surfaces are used. In this case we have 15 in the bottom part of the structure, where the confinement of the field to the surface has to be high. The permittivity value gradually decreases when the height of the cosine object increase.
- Simplicity in fabrication: a discretization process was performed on the continuous distribution to give a very simple seven-layered structure. To this regard, both the surface and the dielectric slab have linear boundaries, and the slab has a constant thickness perpendicular to the surface of the metallic ground plane, as illustrated in Fig. 1(c,d).

The manufacturing technique was used to fabricate three different samples: the Flat Plane (FP), the Uniform Dielectric Surface (UDS), both of them with single relative permittivity of 15, and the Graded Index Surface (GIS), as shown in Fig. 1(e,f).

### Device Fabrication

The cloak was fabricated by layering multiple dielectrics on a cosine-curved metal plate, which would act as ground plane for the device. As per design requirements^13^, the device was discretized into seven distinct layers that varied from a relative permittivity of 9 to 15. The permittivity values have been achieved using alternative dielectric mixtures with differing volume fractions and particle sizes ranging from nanometre to micron ranges.

The materials used are similar to those described in refs 15 and 16, however the fabrication process and challenges are significantly different as in this case the structure design consists of coats of different dielectric films compared to essentially bulk structures. In particular, the device was fabricated using a series of novel techniques, which can be broken down into three distinct stages: particulate filler preparation, composite production and a multi-cast, sequential layer fabrication.

The first challenge was to prepare a casting mixture that would deliver the required permittivity values. The relative permittivity was controlled through tailoring both the particle size and volume fraction. The homogeneous electromagnetic properties (throughout every layer of the composite structure) were achieved through a deep understanding of the relationship among volume fraction, viscosity and curing regime.

Thus, a layering methodology was employed using an open architecture multiple casting route to define each layer precisely. The second challenge was to ensure precise layer dimensional tolerances, e.g. layer thickness, layer width, concentricity, and control of geometric features. The sidewalls of the dielectric layers are angled precisely to match the modelling discretisation requirements. Similarly, the cross-sectional angles of each of the “concentric” underlying discretised annuli of the metallic backplane are precisely formed, in this instance through computer controlled machining.

The optimised mixtures for the discretized layers (each 4.5 mm thick and perpendicular to the lower metal surface) were cast sequentially onto the lower material ground plane. Once cured, the top surface of the structure was machined to match the desired surface profile. This manufacturing technique is advantageous due to the fact that it is inexpensive and highly reproducible. Each layer of the cloak was tailored to the required relative permittivity as dictated by the design process. A list of the achieved electrical properties can be found in Table S1. Optical microscopy characterisation was used to inspect the cured materials to confirm homogeneity and to measure the relative permittivity of the prepared materials between 8 GHz and 12 GHz, exhibiting a stable dielectric constant over this frequency band.

### Numerical and experimental setup

**Figure 2:**
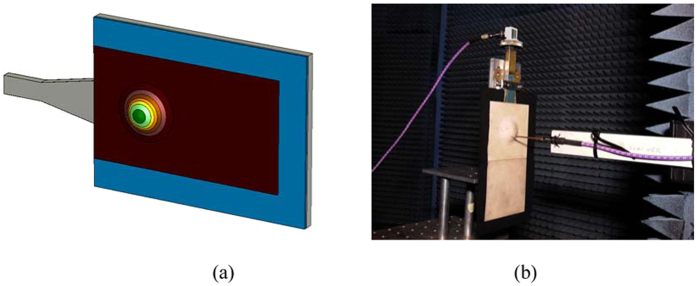
(**a**) Numerical setup for surface wave cloaking device; (**b**) Experimental setup for detecting surface waves on devices.

In terms of excitation, the device is illuminated by an E-plane pyramid horn antenna placed at the left. In an effort to faithfully match laboratory conditions, an actual absorbing layer with real-to-life electrical properties (ε_r_ = 3.8–j7.2 at 10.0 GHz) is used to bound the entire structure in the xy-plane (Fig. 2(a)). In terms of dimensional properties, all the three samples have equivalent foot-prints (140 mm × 140 mm), and dielectric layer thickness (4.5 mm). The two surfaces that contain a deformation have a maximum height of 17.1 mm.

All simulations were solved using a full-wave, commercial, electromagnetic solver (CST 2014). All materials were simulated by using actual manufactured electrical properties (Table S1).

The experimental setup can be seen in Fig. 2(b). An additional flat dielectric substrate is placed next to the deformed surfaces to allow for a greater area of scan. The reason for the attached flat dielectric slab is to ensure that a sufficient amount of space was available in the forward scattering region in order to clearly reveal the performance of the cloak. The mode excitation is provided by a pyramid horn antenna with a central operating frequency of 10 GHz, attached to port 1 of an Agilent N5230C PNA-L network analyser. In order to detect the surface wave along the device, a monopole probe is positioned 0.5 mm above the surface and was attached to port 2 of the PNA-L. With this two-port set up, the S21 parameter was measured at discrete points along the devices (at a resolution of 1 mm × 1 mm) from which the corresponding amplitude and phase of the electric field component normal to the plane (E_z_) were calculated. To scan both the flat and curved regions of the device an NSI planar scanner was pre-programmed to follow the contours of the surface under investigation. The probe was positioned normal to the flat region of the device (z-axis). In doing so, only components parallel to the orientation of the probe can be detected. As mentioned in the numerical setup, both devices were surrounded on their edges by an absorbing layer to reduce reflections at the boundaries.

### Measurements and data processing

**Figure 3:**
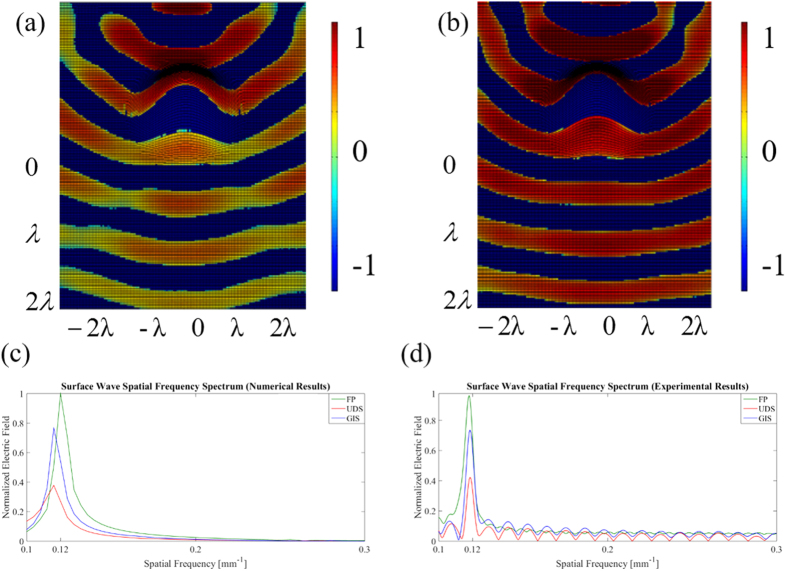
3D measurements of the E-field on the curved surface for (**a**) UDS and (**b**) GIS. Surface Wave Spatial Frequency Spectrum for (**c**) numerical and (**d**) experimental results, at 10.0 GHz.

One of challenges in measuring surface wave components is to decompose them from measured data. The electric field 3D-scanning results for the deformations UDS and GIS on the x-y plane are shown in Fig. 3(a,b), respectively.

Here we consider only the E_z_ component, instead of the full-field representation, due to the fact that analytical and numerical analysis, revealed that the other electric field components (along x and y) can be considered negligible in terms of amplitude distribution, compared to E_z_. (See Supplementary Fig. S1).

Analysis of E_z_ for the back space is omitted for two reasons. First, the amplitude of the E_z_ is many orders of magnitude greater before the bump, owing to the fact that this where the aperture of the horn antenna is located. Secondly, and perhaps more importantly, we are ultimately more interested in forward-scattering performance after the surface deformation.

Here spectral analysis has been used to decompose the complex signals into simpler parts by applying Fourier Transform. The analysis consists in the following steps:

(1) Fourier Transform from spatial domain to frequency spatial domain (normalized k-vectors space), which contains both space and surface waves components (See Supplementary Fig. S2a).

(2) Identification of the space and surface wave phenomena in the bi-dimensional spatial frequency spectrum (See Supplementary Fig. S2b). The normalized wave vector is defined as 
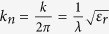
 then at 10 GHz for the free space (being *ε*_*r*_ = 1) *k*_*n*_ = 0.033 mm^−1^ and for the surface wave *k*_*n*_ = 0.12 mm^−1^ (being *ε*_*r*_ = 13.9, see Table S1). It is important to note that the discrepancy between numerical and experimental results, in the spatial frequency spectrum, can be explained by the limited spatial resolution of the scanning system: it is important to point out that the monopole probe itself has an actual diameter of about 1 mm, which also ultimately affects the underlying resolution of the scanning system. In this way, higher-order harmonics of the space wave have been lost in the noise floor of received signals. On the other hand, in the analytical and numerical results, the “scanning” resolution is directly determined by the fineness of the grid used and only limited by computational capacity.

(3) Filtering and isolation of surface wave component: Once we identified and separate the space and surface wave components, we apply a 2D windowed Fast Fourier Transform (FFT) technique (focus only on the surface wave component) to filter out the unwanted space wave (See Supplementary Fig. S2c).

(4) Surface wave conversion in the spatial domain: an inverse transformation is applied to go back to the spatial domain. Thus we obtain the field distribution of the electric field that only contains the surface wave (See Supplementary Fig. S2d).

## Results

Let us consider the scattering problem of a time-harmonic plane wave (*e*^*jωt*^) by an arbitrary object placed at the origin of a spherical coordinate system (*r*, *θ*, *ϕ*). By solving the Helmholtz equation the electromagnetic configuration (in terms of electric field) can be analytically described by^14^:


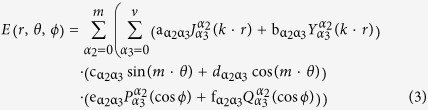


The electric field is a superimposition of Bessel functions (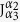 and 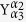 first and second order, respectively), Legendre polynomials (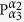 and 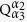 are the associated Legendre functions) and circular functions (Sine and Cosine), where *m* and *v* are the order and degree of such functions, respectively. The coefficients (α_2,3_) quantify how much scattering is associated with a given incident wave and depend only on the particular object (size and shape) and the considered frequency.

In particular, for the FP case the electric field propagates undisturbed along the surface dielectric – air. By applying the appropriate boundary conditions and after some mathematical manipulations, the electric field expression reads^17^:


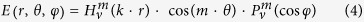


**Figure 4:**
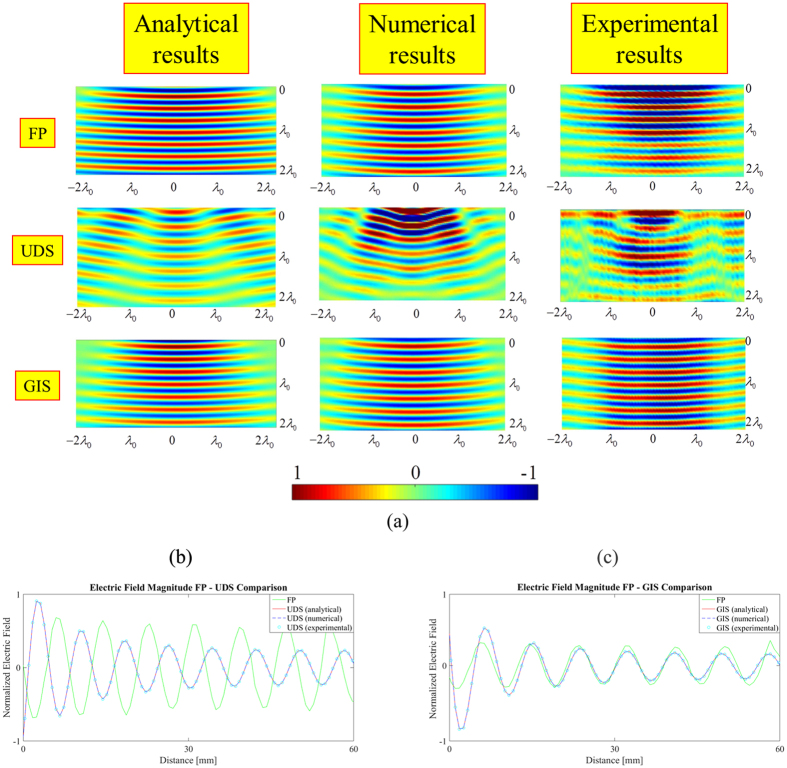
(**a**) Ez (real part) comparison for all the three samples in terms of analytical, numerical and experimental model along the x-y plane; Magnitude (real part) comparison for the normal electric field Ez for (**b**) FP – UDS and (**c**) FP –GIS. (λ_0_ = 30 mm, f_0_ = 10 GHz).

Figure 4(a) (row 1) shows the analytical, numerical and experimental E_z_ component plotted along the x-y plane for the FP case at 10 GHz.

It is obvious that the presence of a metallic object, along the path of the travelling wave, drastically changes the aforementioned configuration. For objects that are very large (when compared with the operative wavelength) the numbers of dominant scattering harmonics rise fast with the size of the object. Therefore the related electric field (real part) can be expressed as:


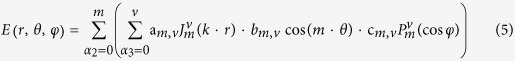


Figure 4(a) (row 2) shows the E_z_ component plotted along the x-y plane for the UDS case at 10 GHz for the analytical, numerical and experimental models. Here we note a small amount of back scattering, mostly owing to the geometrically smooth nature of the surface deformation itself. In addition, a considerable amount of forward scattering is present. It is caused by different path lengths of the wave traversing the curved surface and that one travelling along the flat surface. The net result of this is the creation of a destructive interference pattern on the exiting side of the deformation, or in other words shadowing.

In order to cloak the object, we use the dielectric profile found out in the design process, specifically that one expressed in (1). It ensures, first of all a strong confinement of the surface wave along the interface dielectric-air and secondly (more importantly) the equality of the path lengths (on the curved surface and along the flat one) for the traveling surface wave. In this way, propagation with no backward and forward scattering is achieved.

Figure 4(a) (row 3) shows the analytical, numerical and experimental results for the GIS scenario at 10 GHz. Specifically, compared to the previous case (UDS), there is a pronounced difference in the behaviour of E_z_ in the forward scattering direction. More specifically, there is a noticeable reduction in the amount of shadowing seen immediately after the object, as well as a noticeable improvement in the reconstruction of wave fronts, both of which are promising indicators of an effective surface wave cloak.

The minor “fractures” in the wave fronts at the right and left edges of the scanning region are mostly caused by minor reflections at the interface of the dielectric substrate and the absorbing layer.

In order to quantify the forward scattered field after the filtering process, the normal component of the electric field E_z_ is examined along a sample line (in the radial direction *r*, which length is 60 mm), the results are shown in Fig. 4(b,c). Here a comparison of the E_z_ component for the analytical, numerical and experimental results between FP-UDS and FP- GIS is reported.

**Figure 5:**
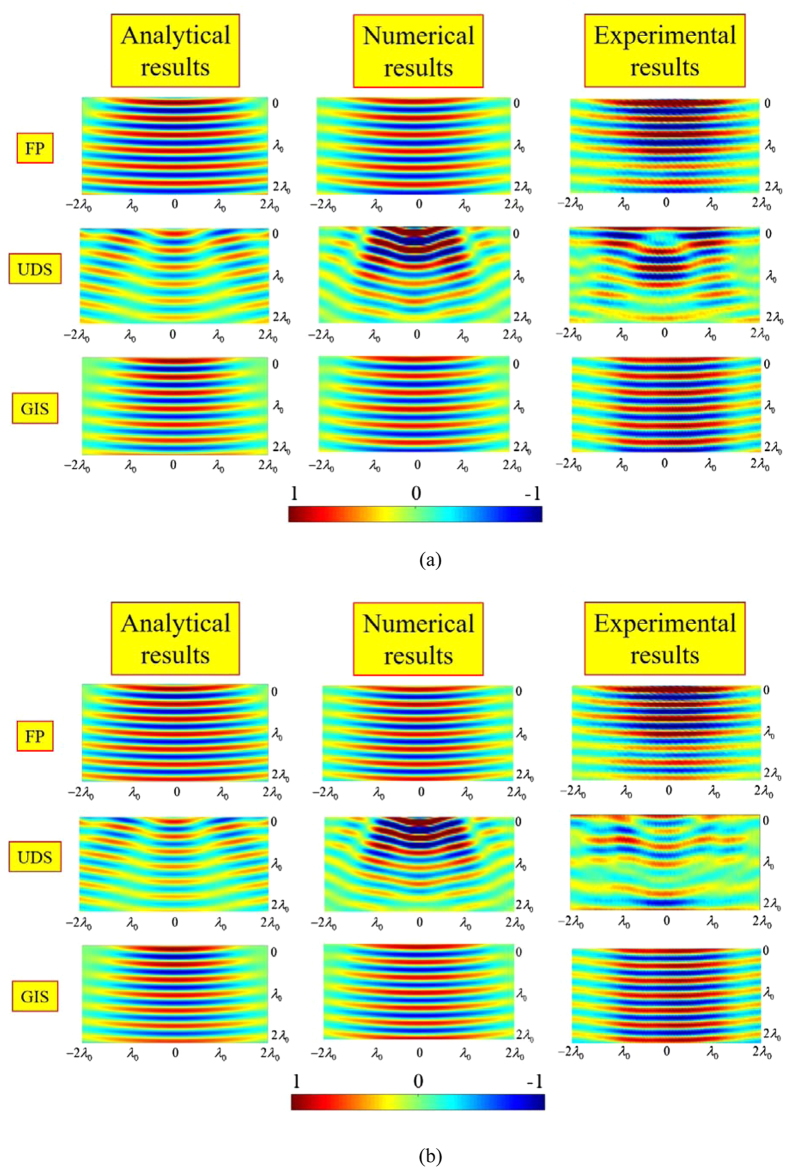
Ez (real part) comparison for all the three samples in terms of analytical, numerical and experimental model along the x-y plane (λ_0_ = 30 mm): at (**a**) 8 GHz and (**b**) 9 GHz.

To demonstrate the bandwidth performance of the device, E_z_ is plotted for the forward scattering region also at 8 and 9 GHz (Fig. 5(a,b), respectively).

## Discussion

In this paper we designed, fabricated, simulated and experimentally validated the performance of an all-dielectric, isotropic and omnidirectional, device for surface wave cloaking. First of all, a transformation optics-based approach was used to design an isotropic graded index media useful to cloak the metallic object underneath. Novel mixing techniques were employed to fabricate the device and to deliver the required permittivity values whilst simultaneously controlling their rheology. Next, the realized electrical properties were imported into the analytical and numerical models, in order to emulate laboratory conditions. A full-wave, commercially available, electromagnetic solver was used to validate the devices performance over the band of interest (8–10 GHz). Lastly, an experimental study was carried out using a microwave near-field scanning system to probe amplitude and phase of the electric field component along the device, which demonstrated that the proposed surface wave cloaking device functions properly. We argue that the proposed technique has much wider applications than the surface wave cloak alone, it can be extended to engineering applications at THz and optics where surface wave radiation and propagation is utilised.

## Additional Information

**How to cite this article**: La Spada, L. *et al*. Surface Wave Cloak from Graded Refractive Index Nanocomposites. *Sci. Rep.* **6**, 29363; doi: 10.1038/srep29363 (2016).

## Supplementary Material

Supplementary Information
