# Supplementary material for: Surface Wave Cloak from Graded Refractive Index Nanocomposites
Source: Sci Rep. 2016 Jul 15;6:29363. doi: 10.1038/srep29363 (PMC4945873; doi:10.1038/srep29363)
Supplement: Supplementary Information [file srep29363-s1.pdf]

Surface Wave Cloak from Graded Refractive Index Nanocomposites

Supplementary Material

L. La Spada[1], T. M. McManus[1], H. Dyke[2], S. Haq[2], L. Zhang[1], Q. Cheng[1], Y. Hao[1]

[1]School of Electronic Engineering and Computer Science, Queen Mary University of London, E1 4NS, England, UK
[2]BAE Systems Advanced Technology Centre, BS34 7QW, England, UK

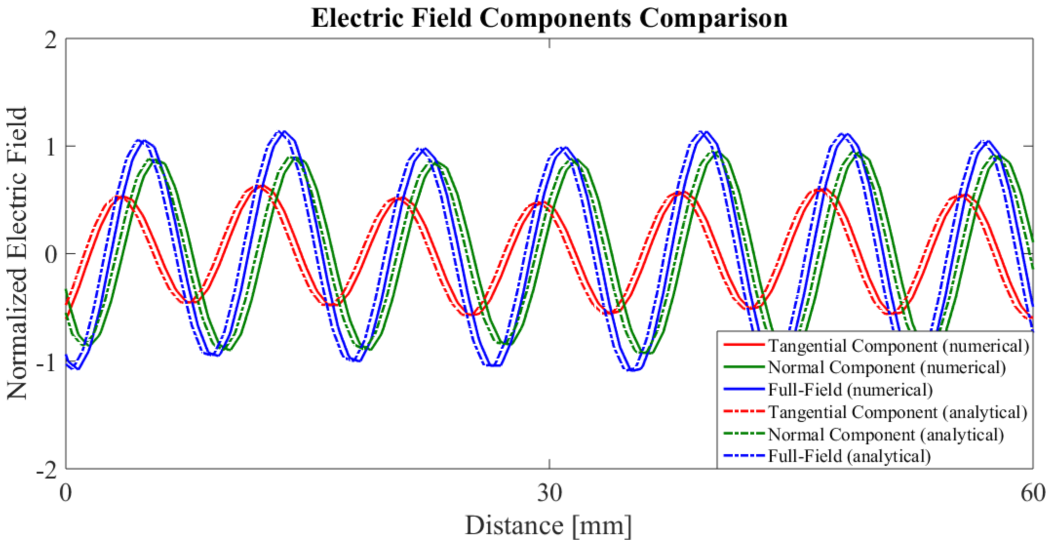

Figure S1: Normal and Tangential components comparison (analytical and numerical results) for the Electric Field

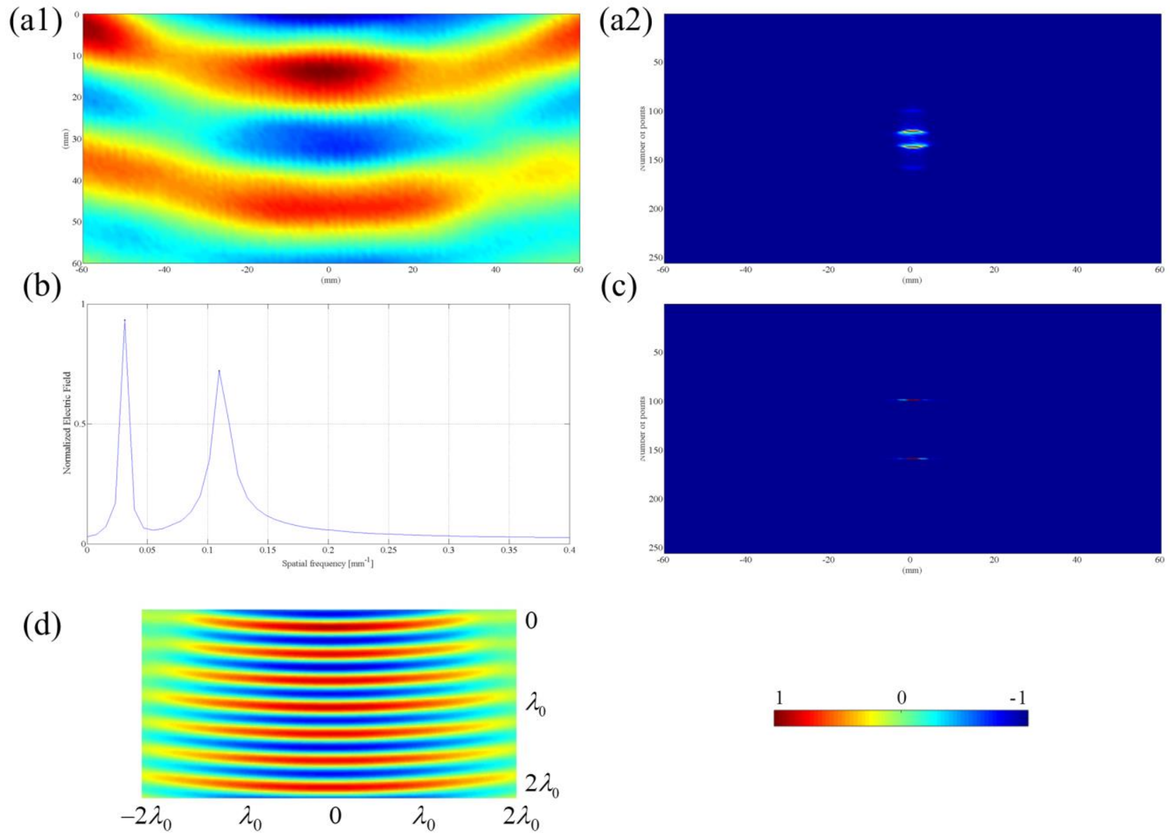

Figure S2: Example of FFT procedure for GIS at 10 GHz (λ_0=30 mm): (a1) Electric Field Measured (before spectral analysis) and (a2) Electric Field 2D Spatial Frequency Fourier Transform; (b) Spatial Frequency Spectrum; (c) Surface Wave Component 2D Spatial Frequency Fourier Transform and (d) Surface wave conversion in the spatial domain.

Desired permittivity	Real permittivity	Imaginary permittivity
9	8.8	0.5
10	9.9	0.6
11	11.1	0.7
12	12.0	0.8
13	12.6	0.9
14	13.7	1.0
15	13.9	1.0

Table S1: Desired permittivity and measured values of real permittivity and imaginary permittivity at 10GHz.
